# Evaluation the effectiveness of the Jiangniaosuan formulation in the treatment of hyperuricemic nephropathy in patients with chronic kidney disease stages 3–4: Study protocol of a randomized controlled trial

**DOI:** 10.1016/j.conctc.2023.101065

**Published:** 2023-01-17

**Authors:** Lili Lu, Li Xu, Yikun He, Jiaying Shen, Jiadong Xin, Jiabao Zhou, Chuanxu Wang, Yating Wang, Xin Pan, Jiandong Gao

**Affiliations:** a Department of Nephrology, Shuguang Hospital Affiliated to Shanghai University of Traditional Chinese Medicine, TCM Institute of Kidney Disease, Shanghai University of Traditional Chinese Medicine, Key Laboratory of Liver and Kidney Diseases(Shanghai University of Traditional Chinese Medicine), Ministry of Education, Shanghai Key Laboratory of Traditional Chinese Clinical Medicine, Shanghai, China; b Department of Rheumatology, Shuguang Hospital Affiliated to Shanghai University of Traditional Chinese Medicine, Shanghai, China

**Keywords:** Hyperuricemic nephropathy, Chronic kidney disease, Traditional Chinese medicine, Febuostat, Randomized controlled trial

## Abstract

**Background:**

Hyperuricemic nephropathy is a highly prevalent kidney disease induced by excessive accumulation and deposition of monosodium urate in kidney, which contributes to the loss of kidney function. The Jiangniaosuan formulation (JNSF) is a Chinese herbal medicine treatment. The aim of this study is to evaluate its efficacy and safety among patients with hyperuricemic nephropathy at chronic kidney disease (CKD) stages 3–4 and with obstruction of phlegm turbidity and blood stasis syndrome.

**Methods:**

Our research is designed as a single-centre, double-blinded, randomized, placebo-controlled trial for 118 patients diagnosed with hyperuricemic nephropathy at CKD stages 3–4 and with obstruction of phlegm turbidity and blood stasis syndrome in mainland China. Patients are to be randomized into two groups: either the intervention group which receives JNSF 20.4 g/day combined with febuxostat 20–40 mg/day, or the control group which receives JNSF placebo 20.4 g/day combined with febuxostat 20–40 mg/day. The intervention will be carried on for 24 weeks. The change in estimated glomerular filtration rate (eGFR) is set as the primary outcome. Secondary outcomes include changes in serum uric acid, serum nitric oxide, urinary albumin/creatinine ratio, urinary *N*-acetyl-β-D glucosaminidase, urinary β2 microglobulin, urinary retinol binding protein and TCM syndromes in 24 weeks. Statistical analysis will be formulated by SPSS 24.0.

**Discussion:**

The trial will conduce to the comprehensive assessment in the efficacy and safety of JNSF among patients diagnosed with hyperuricemic nephropathy at CKD stages 3–4, and provide a clinical method available on systems of the combination of modern medicine and TCM.

## 1 Background

Hyperuricemic nephropathy (HN) is a pathological condition induced by hyperuricemia (HUA) or gout. Hyperuricemia is caused either because of the excessive production or decreased excretion of uric acid (serum uric acid >0.41 mmol/L or 6.8 mg/dL) [1]. Classically, the accumulation of monosodium urate crystals in kidney tissues cause inflammatory reactions that contribute to kidney damage. The incidence of gout is in the range of 0.6–2.9/1000 individuals per year worldwide, and the adult prevalence ranges from 0.68% to 3.90% [2]. The prevalence rate of gout in Chinese adults is 1.1%; a trend of increase has been observed from 1.0% in 2000–2005 to 1.3% in 2010–2016 [3]. From 2000 to 2014, the aggregate prevalence has been approximately 13.3% and 1.1% in patients with hyperuricemia and gout in mainland China [4]. The increase in the incidence of hyperuricemia and gout has been accompanied by an increase in the number of patients with hyperuricemic nephropathy. This poses a serious threat to health of individuals. Approximately 100% of patients with gout exhibit renal damage during autopsy [5]. In general, hyperuricemic nephropathy is a frequent occurrence observed in different types of progressive chronic kidney disease (CKD). The expensive charge for hemodialysis and kidney transplantation results in a tremendous financial burden both to individuals and society. Therefore, it is particularly important to focus on the delay of the further progression of CKD.

At present, the conventional treatment for hyperuricemic nephropathy mainly involves the administration of urate-lowering therapy (ULT) and symptomatic treatment of complications, adverse events, and toxic side effects. Xanthine oxidase inhibitors (XOI) are considered first-line ULT [6], including allopurinol and febuxostat. However, either of them did not retard the decline of eGFR in comparison with placebo in CKD stages 3–4 patients [7,8].

Currently, several treatment alternatives, including traditional Chinese medicine (TCM), which is a supplementary therapy for hyperuricemic nephropathy, are available. During the treatment with TCM, phlegm turbidity stasis resistance is considered to be the main pattern observed in stages 3–4 (advanced stage) CKD patients with hyperuricemic nephropathy based on syndrome differentiation. The Jiangniaosuan formulation (JNSF) contains seven herbs, i.e., 10 g Semen Vaccariae, 10 g Semen sinapis, 15 g Semen plantaginis, 10 g Dioscoreae hypoglaucae rhizoma, 10 g Fructus crataegi, 15 g Radix clematidis, and 10 g Radix et rhizoma rhei. JNSF is mainly used to dissolve phlegm and reduce turbidity, promote blood circulation, and promote collaterals, which is in accordance with the pattern observed in patients at advanced stages of hyperuricemic nephropathy.

Our previous work has demonstrated that JNSF can lessen urate crystallization, suppress renal inflammation and improve renal function in rats with hyperuricemic nephropathy, and the changes could be contributed to suppressing the TLR4/NF-κB signaling pathway [9]. However, no evidence-based clinical study has appraised the efficacy and safety of the clinical application of JNSF for the therapy of hyperuricemic nephropathy. Consequently, we conduct a randomized, double-blinded, placebo-controlled clinical approach to assess whether clinical efficacy could be improved with the use of JNSF for the treatment of stages 3–4 CKD patients with hyperuricemic nephropathy. This would also provide an effective approach and help us accumulate evidence-based medical support for the treatment of hyperuricemic nephropathy in stages 3–4 CKD patients using traditional Chinese medicine.

## 2 Methods and design

### 2.1 Trial design

**Fig. 1:**
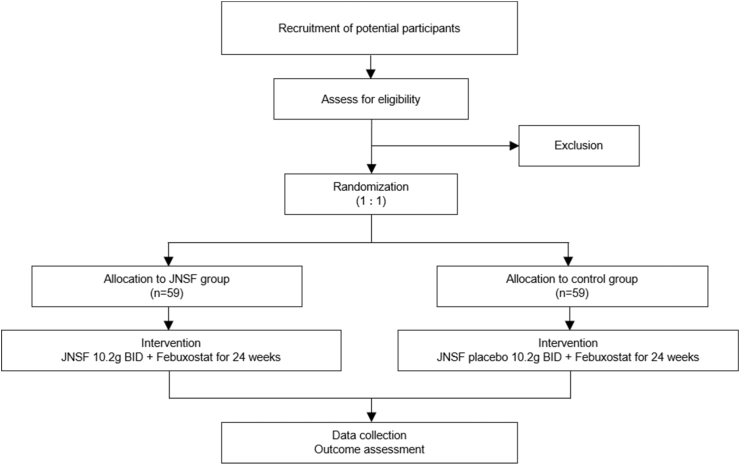
Flowchart showing the process to be followed for the JNSF study during the intention-to-treat analysis.

This study will be a prospective, single-centre, double-blinded, randomized, placebo-controlled trial involving stages 3–4 CKD patients with hyperuricemic nephropathy from mainland China. Patients are to be randomized into either the Jiangniaosuan formulation (JNSF) and febuxostat group or the JNSF placebo and febuxostat group. The intervention will be carried on for 24 weeks. This is designed as a superiority trial with allocation ratio = 1/1. The design of this study is laid out in Fig. 1.

### 2.2 Ethics and registration

This study protocol (Version 2021.01.20.01) has been approved by Ethics Committee of Shuguang Hospital affiliated with Shanghai University of TCM (Approval Number: 2021-942-17-01). The trial has been registered with the Chinese Clinical Trials Register (ChiCTR2100049048) on July 20, 2021. The chief investigators are those clinicians who have acquired relevant qualifications. Before, during or after the trial, the personal data and biospecimens of enrolled participants will not be revealed to any third party. The results will be disseminated through scientific meetings and published in peer-reviewed journals.

### 2.3 Participating centre

The study will recruit 118 outpatients or inpatients from the Department of Nephrology or Rheumatology at Shuguang Hospital affiliated to Shanghai University of Traditional Chinese Medicine.

### 2.4 Study population

We will enroll 118 stage 3–4 CKD patients diagnosed with hyperuricemic nephropathy with phlegm-turbid obstruction and blood stasis syndrome.

### 2.5 Sample size

The sample size is issued from the primary outcome that is an alteration in baseline estimated glomerular filtration rate (eGFR). According to a previous report [8], the average eGFR value of the control group is estimated to be 34.7 mL/min/1.73 m^2^. After treatment with JNSF, the value for the intervention group could be increased by 10 mL/min/1.73 m^2^, and its standard deviation would be approximately 18.1 mL/min/1.73 m^2^. If we set the type 1 risk level at 5% and the power at 80%, we calculated that the minimum sample size for each group is 53 participants, using the formula: $t=\frac{\bar{x_{1}}-\bar{x_{2}}}{\sqrt{\frac{\left(n_{1}-1\right)s_{1}^{2}+\left(n_{2}-1\right)s_{2}^{2}}{n_{1}+n_{2}-2}\left(\frac{1}{n_{1}}+\frac{1}{n_{2}}\right)}}$. Assuming a drop-out rate of approximately 10%, we determined that the sample size should be increased to 59 in both groups, to include a total of 118 patients.

### 2.6 Randomization procedures

Potential participants will be screened for eligibility a week anterior to study randomization. Participants will be randomly assigned as a consequence of a randomization schedule to receive JNSF treatment coupled with febuxostat (intervention group) or JNSF placebo treatment coupled with febuxostat (control group).

These treatments are changed into two groups A and B by the independent statistician. A random numbering (1:1) is formulated based on complete randomization with a block size of 4 via SPSS software.

### 2.7 Blinding

This trial will be conducted in a double-blinded manner. The allocation process is blind to all individuals, including the physicians, clinical trial pharmacists, investigators, and patients, apart from the independent statisticians as they generate the random numbering.

The placebo granules and the JNSF granules will be tantamount in the matter of packaging, shape, color, and taste. The pharmaceutical company will pre-paste consecutive random numbers on the package based on the randomization schedule dispatched from the independent statistician. Patients will be given the package of JNSF or the placebo in the order of their visit by clinical trial pharmacists. The double-blind trial is divided into a primary unblinding and a secondary unblinding after the end of the last patient follow-up. The first level of unblinding is to reveal whether each patient belongs to group A or B and to facilitate comparisons between the groups by the statistician. After the statistical conclusions are obtained, a secondary unblinding is required to be informed of the medication use in each group. The independent statistician will provide the principal investigator with a set of sealed and externally numbered envelopes, which inside is the medication used. In case the serious adverse events (SAEs) occur, early broken blinding is performed by the principal investigator to estimate whether there is a connection to the intervention drug, overdose, serious drug interactions with combined drugs or the urgent need to adopt a resuscitation plan.

### 2.8 Eligibility criteria

#### 2.8.1 Diagnostic criteria for hyperuricemic nephropathy

According to the diagnosis, syndrome differentiation, and curative effect in patients with hyperuricemic nephropathy (trial Scheme) [10], a patient must fulfill the requirements of the third item, and at least one of the five other items listed below, in order to be diagnosed with hyperuricemic nephropathy.

(1) The patient should be a middle-aged male or post-menopausal female who has often had gouty arthritis, gout nodulosis, or uric acid stones in the urinary trac.
(2)The patient should demonstrate clinical manifestations of chronic interstitial glomerulonephritis, including mild to moderate proteinuria or decreased urinary concentrations (low osmotic pressure in the first-morning urine) in the early stage, and hypertension and azotemia in the later period.
(3)Serum uric acid levels should be > 357 μmol/L for females and >417 μmol/L for males.
(4)Urine sediment examination results should demonstrate uric acid crystallization, microscopic or gross hematuria, and pyuria.
(5)Urine sediment examination results should demonstrate uric acid crystallization, microscopic or gross hematuria, and pyuria.
(6)Renal histology is mainly manifested as renal tubule-interstitial lesions, and birefringent needle-like urate crystals are found in the renal interstitium and renal tubules.

#### 2.8.2 Diagnostic criteria for patients with stages 3–4 of CKD

Recruited male and female patients should exhibit abnormal laboratory test results or anomalies of renal structure and a loss of endocrine function beyond three months. Patients should be diagnosed with an eGFR value ≥ 15 mL/min/1.73 m^2^ and <60 mL/min/1.73 m^2^. The eGFR should be computed utilizing the chronic kidney disease epidemiology collaboration (CKD-EPI) creatinine equation.

#### 2.8.3 Diagnostic criteria for TCM syndrome differentiation

Based on an evaluation of the diagnosis, syndrome differentiation, and curative effect in patients with hyperuricemic nephropathy (trial Scheme) [10], a patient, to be diagnosed with the obstruction of phlegm turbidity and blood stasis syndrome, has to perform no less than three of the primary symptoms listed below.

Primary symptoms.

◆ Pain or swelling in the joints
◆Joint stiffness and deformation, possibly with subcutaneous gout nodules
◆Impaired flexion and extension of joints
◆Fatigue and swelling limbs
◆Sticky and greasy in the mouth
◆Increased nocturia
◆Frequent urination
◆Waist sinking and pain
◆A red or dark tongue with ecchymosis
◆A yellow and greasy coating of the tongue
◆A string-like and slippery pulse

Inclusion criteria and exclusion criteria are listed below (Table 1).

**Table 1:** Eligibility criteria for the trial.

Inclusion criteria
To be eligible for participation patients must be:
◆ Individuals diagnosed with hyperuricemic nephropathy beyond three months as listed above, at the same time diagnosed with obstruction of phlegm turbidity and blood stasis syndrome in TCM
◆ Aged between 18 and 75 years
◆ The eGFR value ≥ 15 mL/min/1.73 m^2^ and <60 mL/min/1.73 m^2^
◆ Should be able to provide serum or urine pregnancy test results (for females), 24 h earlier than JNSF administration
◆ Should be able to provide the investigators with informed consent in written form for trial enrollment
**Exclusion criteria**
Patients are to be unentitled for participation if:
◆ The patient is probably or distinctly possible or definitely allergic to any of the components of the intervention or control drugs
◆ 24-h proteinuria value > 1 g
◆ The patient is diabetic
◆ The patient has had unresolved acute kidney injury, nephrotic syndrome, inherited kidney diseases, another serious disease, or renal transplantation in the previous 3 months
◆ The patient disagrees to engage in the study
◆ The patient has chronic hepatitis, or hepatic dysfunction induced by drug or other pathogens
◆ The patient has recently participated in another clinical trial
◆ The patient is taking medications as similar with JNSF
◆ The patient has a complication or history of malignant tumors, while made an exception for that the malignant tumor is untreated and not recurred within 5 years
**Rejection criteria**
Patients will be eliminated if they correspond with one of the rejection criteria:
◆ Do not meet inclusion criteria or meet exclusion criteria
◆ Vacate from informed consent
◆ If no next-in-line statistics are acquired after admission
◆ Commit a serious violation of trial rules, including receiving other therapies concurrently to such an extent that efficacy and safety could be indistinguishable
**Drop-out and Termination criteria**
Patients are to be terminated on condition that they correspond with one of the following criteria:
◆ The participant withdraws from the trial (because the patient becomes invalid, lost, or dies accidently)
◆ The interviewee is terminated during the trial (for reasons such as poor compliance, withdrawal of informed consent, prohibition of drug use, or safety-related problems)
◆ When SAEs or other diseases occur, it is not appropriate to continue to conduct
◆ Incomplete data affects the evaluation of efficacy and safety
◆ A serious violation of trial procedures has occurred
◆A hepatocellular injury is detected because of the abnormally elevated liver enzymes: level of alanine transaminase (ALT) or aspartate transaminase (AST) is more than three times of the upper limit of normal (ULN)

### 2.9 Manufacture of study supplements

Patients will be randomized into either the JNSF or the control group and orally administered JNSF or placebo. Patients in the JNSF group will receive 10.2 g of the allocated intervention drug JNSF granules twice a day as oral administration, 30 min later than breakfast and dinner lasting 24 weeks. Respectively, the control group will accept 10.2 g of the placebo twice a day as oral administration. The appearance of the placebo is consistent with that of JNSF granules, in terms of packaging, appearance, particle shape, color and taste, but it contains one-tenth of the volume of drugs in the JNSF granules. The JNSF granules, as well as the placebo granules will be produced by Jiangyin Tianjiang Pharmaceutical Co., Ltd. (Wuxi City, Jiangsu Province, China), in accordance with certain manufacturing practices. The composition and function of each herb of JNSF have been outlined in Table 2.

**Table 2:** Composition, effects and pharmaceutical action of the Chinese herbal medicines that constitute JNSF.

Ingredient	Granule dose	Effect (TCM)	Pharmaceutical action
Semen Vaccariae (wangbuliuxing)	10g	Promotes blood circulation and removes obstruction in collaterals	Attenuates human endothelial cell oxidative stress injury
		Promotes lactation for resolving carbuncle	Inhibits the growth and metastasis of malignant cells
Semen sinapis (baijiezi)	10g	Dissipates phlegm and expels retained fluid	Suppresses the mRNA expression of a panel of inflammatory mediators
		Resolves masses and removes edema	
Semen Plantaginis (cheqianzi)	15g	Reduces heat and induces diuresis	Reduces the content of urinary protein and UA and deregulates the expression of inflammatory mediators
		Eliminates dampness and frees strangury	
Rhizoma Dioscoreae hypoglaucae (fenbixie)	10g	Promotes diuresis and removes turbidity	Prevents oxidative stress and reduces levels of advanced glycosylation end products in the kidney
		Dispels pathogenic wind and dredges channel blockade	
Fructus crataegi (shanzha)	10g	Resolves food stagnation and invigorates the stomach	Improves lipid metabolism in the serum, liver, and adipose tissue
		Activates qi for resolving blood stasis	
Radix clematidis (weilingxian)	15g	Dispels pathogenic wind and eliminates dampness	Inhibits iNOS gene expression
		Removes obstruction in collaterals for relieving pain	
Radix et rhizoma rhei (dahuang)	10g	Removes heat and dredges the intestine	Anti-renal fibrosis
		Cools the blood and removes toxicity	
		Eliminates blood stasis for dredging channels	Increases the level of serum HIF-1α

iNOS, inducible nitric oxide synthase; HIF-1α, hypoxia inducible factor-1.

Each group will be provided with diet instructions [11], behavioral guidance and health-related education [12]. In addition, they will receive febuxostat as a urate-lowering therapy. The dose of febuxostat should be 20 mg once a day after study initiation to week 4, and this should be increased to 40 mg once a day at week 5 if the target is not met, and maintained until week 24.

Based on KDIGO Clinical Practice Guideline for CKD, medical decisions will be arranged reasonably for patients in either group as basic treatment on how to treat hypertension [13], renal anemia [14], CKD-MBD [15], lipid management [16], concurrent infections, as well as other diseases, with the exception of the TCM herb decoction. The investigators will record the name and dosage of any administered medicine.

### 2.10 Measurement items and procedure

Several relevant examinations will be conducted before the baseline, at the baseline, at week 4, 12, and 24 during treatment. General information will be collected, including the name, age, sex, height, weight, contact information, physical examination, laboratory examination results, and history of present illness, other diseases, personal life, menstruation, marital and fertile, allergy, and family members.

### 2.11 Primary outcome

The primary outcome is the eGFR value, and it is calculated using the CKD-EPI creatinine equation, to evaluate the kidney function before and after the intervention on visit 1 to 4 (listed in Table 3).

**Table 3:** The measurement items and study procedure.

Items	Screening	Treatment			
Timepoint	V0(-1 weeks)	V1(0 week)	V2(4 weeks)	V3(12 weeks)	V4(24 weeks)
Informed consent	X				
Inclusion/exclusion criteria	X				
Demographic data	X				
Medical history	Χ				
Drug combination	Χ	Χ	Χ	Χ	Χ
Randomization		Χ			
Allocation		Χ			
Physical examination	Χ	Χ	Χ	Χ	Χ
TCM symptoms	Χ	Χ	Χ	Χ	Χ
Kidney function (eGFR, Scr, BUN, SUA)	Χ	Χ	Χ	Χ	Χ
Serum electrolytes	Χ	Χ		Χ	Χ
24-h urine test	Χ	Χ		Χ	Χ
UACR	Χ	Χ		Χ	Χ
Renal tube function	Χ	Χ		Χ	Χ
Inflammatory mediators		Χ		Χ	Χ
Renal ultrasound	Χ	Χ			Χ
Blood, urine and stool routine	Χ	Χ		Χ	Χ
Hepatic function	Χ	Χ		Χ	Χ
Electrocardiogram	Χ	Χ		Χ	Χ
Adverse events			Χ	Χ	Χ
Drug distribution		Χ	Χ	Χ	
Drug collection			Χ	Χ	Χ

X indicates that data need to be collected during the visit.

TCM, traditional Chinese medicine; eGFR, estimated glomerular filtration rate; Scr, serum creatinine; BUN, blood urea nitrogen; SUA, serum uric acid; UACR, urine microalbumin to creatinine ratio.

### 2.12 Secondary outcomes

1 Biological indicators: Serum creatinine (Scr), serum uric acid (SUA), blood urea nitrogen (BUN), serum nitric oxide (NO), xanthine oxidase (XOD), 24 h proteinuria, 24 h urine uric acid, urine microalbumin to creatinine ratio (ACR), urinary β2 microglobulin (β2-MG), urinary *N*-acetyl-β-D glucosaminidase (NAG), and urinary retinol-binding protein (RBP) levels will be tested in the clinical laboratory.
2 Inflammatory mediators: Blood plasma levels of interleukin 1β (IL-1β), interleukin 18 (IL-18), and monocyte chemoattractant protein 1 (MCP-1) will be measured as they are indicative of systemic inflammation, whereas the protein and mRNA expression levels of NLRP3 and Caspase-1 will be measured to assess the inflammatory characteristics of peripheral blood mononuclear cells (PBMCs). Plasma and serum samples will be stored at - 80 °C.
3 The scores for the curative effect associated with TCM syndromes.

### 2.13 Measurement scale for TCM symptoms

We will apply the measurement scale for TCM symptoms based on the Guidelines for Clinical Research of Chinese Medicine (New Drug) [17], and score a value of 0, 1, 2, or 3 for each symptom indispensable to the diagnosis of the obstruction of phlegm turbidity and blood stasis syndrome, to sum up to yield a total score for each patient. Any change in this score will be formulated an efficacy indicator (EI), which will be used to assess the treatment efficacy:

EI = (Total symptom score at baseline - Total symptom score post-treatment)/Total symptom score at baseline × 100%

The extent of enhancement in symptoms is to be revealed in four categories: full recovery (EI ≥ 90%), marked recovery (90% > EI ≥ 70%), moderate recovery (70% > EI ≥ 30%), and ineffective therapy (EI < 30%).

### 2.14 Data and safety monitoring

The investigators will receive unified training and data monitoring committee (DMC) will be set up with experienced physicians and statisticians, who have no competing interests, to conduct monitoring once every three months for the entire research process. The trained investigators will fill out the case report form (CRF), and use EpiData 2.1a software to establish the special database system for this trial according to the items in the CRF. Two independent data administrators will type the data and conduct audits to ensure that the data in the database is consistent with the results in the CRF.

The participant will be monitored in the circumstance that an adverse event (AE) occurs, until their AE disappears. As patients diagnosed with Hyperuricemic Nephropathy frequently suffer from gout attacks, we will advise patients to oral intake anti-inflammatory drug or low-dose corticosteroids until the pain is relieved. AEs will be reported to the principal investigator to assess and the IRB. The investigator will conduct a follow-up interview and record the results supposing that the intervention induces the AE. Safety monitoring results will be reported to principal investigator weekly and the IRB.

The following are potential events related to the safety of the intervention.

◆ The eGFR is decreased by ≥ 50%
◆ The serum creatine levels is doubled than the previous blood test
◆ The patient is in the progression of CKD stage 5
◆ The patient occurs serious cardiovascular or cerebrovascular event
◆ The heart rate of patient is less than 40 bpm
◆ The patient occurs an unexpected event that is supposed to the intervention by the physicians

The following are potential SAEs.

◆ The patient occurs fatal or life-threatening complications
◆ The patient is dead or persistent and significant disabled
◆ The patient is hospitalized or receive large-scale medical intervention to prevent progression
◆ The patient develops any event that physicians diagnose to be significantly hazardous or harmful

The trial can be terminated for the following reasons.

◆ The number of enrolled patient is insufficient
◆ The allocation codes are unwrapped or the process of blinding has been nullified if the proportion of unsealed envelopes with emergency plans is more than 20% during the trial
◆ SAEs are supposed to associated with the intervention drug in the trial, which may signify that the safety of the drug has serious flaws

### 2.15 Statistical analysis

The independent statisticians will formulate a formal and detailed plan for statistical analysis prior to locking the database. Comparing the baseline demographic data of each group will use with a χ^2^ test or Student's t-test. The means ± standard deviations or medians will express continuous variables, and an independent *t*-test or the Wilcoxon rank sum test will be used to analyze differences of such variables. Repeated measures ANOVA will be built for these outcomes. The numbers and percentages will express categorical variables, and the Fisher exact test or the Mann-Whitney *U* test will be used to calculate and compare differences in such variables respectively. The missing data will be handled with multiple imputation. According to the intention-to-treat principle, all patients enrolled will be embraced in the primary analysis. An independent statistician will use SPSS 24.0 to perform the statistical analysis. Calculations will be performed using GraphPad Prism 6.01 software.

### 2.16 Patient and public involvement

The public and patients are out of circulation with the design, or conduct, or reporting, or dissemination plans of this research.

## 3 Discussion

Traditional Chinese medicine which can improve renal function and reduce uric acid levels is widely used for the treatment of hyperuricemia-associated diseases in China. According to TCM theory, the obstruction of phlegm turbidity and blood stasis syndrome majorly represent the pathogenesis of advanced hyperuricemic nephropathy. The dissipation of phlegm and removal of blood stasis are the main approaches for its treatment. As described previously, the main mechanism of action of JNSF is that it resolves phlegm, reduces turbidity, promotes blood circulation, and promotes collaterals, which is in accordance with the pattern observed for patients at advanced stages of hyperuricemic nephropathy. Moreover, as discussed in our previous study, we found that JNSF can lessen urate crystallization, suppress renal inflammation and improve renal function in a rat model of hyperuricemic nephropathy, and identified its mechanism of action. However, in TCM, syndrome differentiation is mostly derived from empirical medicine and there is a deficiency of convincing evidence. Consequently, we conduct a trial to enroll 118 patients with stages 3–4 CKD and hyperuricemic nephropathy.

Evidence-based medicine (EBM) forms the basis of this study, and each indicator used in the criteria could be quantified in modern medicine. To ensure the use of high-quality methodology and strictly adhere to quality control requirements, we have developed this protocol using the SPIRIT checklist [18]. The accomplishment of this trial might highlight evidence concerning the effectiveness and safeness of JNSF to treat stages 3–4 CKD patients with hyperuricemic nephropathy. We classify the patients into two groups: JNSF plus febuxostat versus JNSF placebo plus febuxostat. JNSF may not be as potent as febuxostat in lowering the uric acid levels, but is advantageous for improving the renal function, according to our clinical observations. Therefore, the primary outcome will be observed in terms of eGFR instead of a decline in uric acid levels. To evaluate the safety, with the exception of kidney function, several parameters will be measured and monitored before and after treatment, including the hepatic function, electrolyte levels, blood and stool routine, blood pressure, heart rate, and electrocardiogram-related differences.

Certain shortcomings are associated with this study. Firstly, the placebo contains excipients such as maltodextrin, caramel pigment, lemon yellow pigment, sunset yellow pigment, and a bittering agent. Theoretically, the use of placebos does not result in therapeutic effects in patients with hyperuricemic nephropathy. However, if these excipients demonstrate a therapeutic effect on patients with hyperuricemic nephropathy, this data has not been recorded. Secondly, in this trial, the age limits are set at 18–75 years, because of the following reasons: older people have multifarious concomitant diseases that increase the difficulty and risk of detection. Besides, we have taken the issue of patient compliance into consideration on the grounds that participants in this trial are mostly outpatients. The onset of hyperuricemic nephropathy with decreased kidney function occurs mostly in adults. Considering our limitations, we narrow down the age range to 18–75 years.

In summary, there are insufficient clinical researches available on systems of the combination of modern medicine and TCM to assess the efficacy and safety to treat patients with hyperuricemic nephropathy. This study will be a combination of an evaluation system for TCM and modern pharmacological researches, and its findings shall serve as a basis for future research in this field. The most prominent feature of this study is to understand TCM classification systems and TCM treatment characteristics based on syndrome differentiation in a more effective manner through the medium of EBM.

## Authors’ contributions

LLL and LX contributed equally to this work. LLL and LX participated in the design of the study, sample size calculation and drafted the manuscript. YKH, JYS and XP are responsible for participants identification and screening. JDX, JBZ, CXW and YTW are responsible for the data collection and analysis. JDG is the principal investigator and contributed to study conception, developing the protocol, and reviewing the manuscript. All authors read and approved the final manuscript.

## Funding

The research is supported by 10.13039/501100003399Science and Technology Commission of Shanghai Municipality (20Y21901800), Key Disciplines Group Construction Project of Pudong Health Bureau of Shanghai (PWZxq2017-07) and the National Natural Science Foundation of China (Grant nos. 81874437 and 81904126). This funding source had no role in the design of this study and will not have any role during its execution, analysis, interpretation of the data, or decision to submit results.

## Declaration of competing interest

The authors declare that they have no known competing financial interests or personal relationships that could have appeared to influence the work reported in this paper.

## Appendix A Supplementary data

Multimediacomponent 1 Multimediacomponent 1

Multimediacomponent 2 Multimediacomponent 2
