# Supplementary material for: Evaluation the effectiveness of the Jiangniaosuan formulation in the treatment of hyperuricemic nephropathy in patients with chronic kidney disease stages 3–4: Study protocol of a randomized controlled trial
Source: Contemp Clin Trials Commun. 2023 Jan 17;32:101065. doi: 10.1016/j.conctc.2023.101065 (PMC9922735; doi:10.1016/j.conctc.2023.101065)
Supplement: Multimediacomponent 1 [file mmc1.docx]

The JNSF is produced by Jiangyin Tianjiang Pharmaceutical Co., Ltd. into granules according to specific production specifications, batch number: 2104322, specification: 5.1g/bag, usage and dosage: 2 times a day, 2 bags each time, taken with boiled water. Valid until March 2024.

Preparation process of granules:

Preparation process of single-flavor extract: take the decoction pieces, add water to decoct twice, combine the decoction, filter, concentrate to a certain relative density of clear paste, spray dry, sieve, and mix.

Mixing: take the 7 flavors of the above extracts according to the prescription, and mix them for 30 minutes to make them uniform;

Granulation: dry granulate the above mixture to make 12-40 mesh granules;

Coding: use blank aluminum foil bags for printing and packaging;

Inner packaging: pack the granules into aluminum foil sachets according to specifications;

Outer packaging: outsource according to specifications, usage, dosage, batch number, validity period and precautions.

Composition of the Chinese herbal medicines that constitute JNSF

Semen Vaccariae (wangbuliuxing)炒王不留行

Semen sinapis (baijiezi)炒芥子

Semen Plantaginis (cheqianzi)盐车前子

Rhizoma Dioscoreae hypoglaucae (fenbixie)粉萆薢

Fructus crataegi (shanzha)山楂

The content is titrated, no spectrum.

Radix clematidis (weilingxian)威灵仙

Radix et rhizoma rhei (dahuang)熟大黄
